# *Syzygium jambos* L. Alston: An Insight Into its Phytochemistry, Traditional Uses, and Pharmacological Properties

**DOI:** 10.3389/fphar.2022.786712

**Published:** 2022-01-24

**Authors:** Melvin Adhiambo Ochieng, Widad Ben Bakrim, Gabin Thierry M. Bitchagno, Mona F. Mahmoud, Mansour Sobeh

**Affiliations:** ^1^ School of Agriculture, Fertilization, and Environmental Sciences (ESAFE), Mohammed VI Polytechnic University, Ben-Guerir, Morocco; ^2^ AgroBioSciences, Mohammed VI Polytechnic University, Ben-Guerir, Morocco; ^3^ African Sustainable Agriculture Research Institute (ASARI), Mohammed VI Polytechnic University (UM6P), Ben-Guerir, Morocco; ^4^ Department of Pharmacology and Toxicology, Faculty of Pharmacy, Zagazig University, Zagazig, Egypt

**Keywords:** *Syzygium jambos*, medicinal plants, pharmacological activities, antioxidant, antiinflammatory

## Abstract

Medicinal plants have been used since ancient times for human healthcare as drugs, spices, and food additives. The progress in technology and medicine observed, the last decades, has improved the quality of life and healthcare but with worrisome drawbacks. Side effects caused by synthetic drugs for instance originate sometimes irreversible health disorders. Natural substances, in contrast, are biologically and environmentally friendly. *Syzygium jambos* L. (Alston) also known as rose apple conveys a long history as essential traditional medicine with a broad spectrum of application in various cultures. The plant discloses a diverse group of secondary metabolites and extracts that displayed major susceptibilities towards various health concerns especially stress-related and inflammatory diseases. Despite a rich literature about the plant, the chemistry and biology of *S. jambos* have not been comprehensively reviewed yet. Accordingly, we present herein a literature survey of rose apple which aims to draw the chemical identity of the plant and establish a consistent discussion on the respective biological application of plant extracts and their corresponding traditional uses. The present work could provide a scientific basis for future studies and necessary information for further investigations of new drug discovery.

## Introduction

The renown of alternative medicines nowadays is appealing although progress in technology and medicine encountered the last decades has improved the quality of life and healthcare around the world. Corresponding drawbacks are quite worrisome. Side effects caused by synthetic drugs for instance hurt human health system, sometimes with irreversible impacts (van Wyk and Wink 2015). Natural substances, in contrast, are biologically and environmentally friendly as they are recognized by other organisms which facilitate their metabolisms. These substances are provided from plants, microorganisms, or animals with a pronounced interest since they constitute the main sources of foods and thus, our first resort in case of pain (van Wyk and Wink 2015).

Plants contain chemicals not essential for their metabolism rather for the fight against attacks and stress due to the plant habitats. These phytochemicals have shown distinct biological properties against numbers of illnesses (Iwu, 1993; van Wyk and Wink 2015). Both plants and compounds are of great interest in drug development to face new medical challenges.

Accordingly, numerous of research works have been conducted on plants from the genus *Syzygium* to elucidate its chemistry and pharmacology. Species of this genus, including *S. jambos,* offer edible fruits found under various formulation including juices, jellies, and jams (Sun et al., 2020). The decoction of these fruits serves to alleviate gastrointestinal disorders, wounds, syphilis, leprosy, as well as toothache (Chua et al., 2019). Reports have highlighted the occurrence of polyphenols, flavonoids, tannins, and sterols from various organs of *S. jambos* species. Meanwhile, plant extracts and compounds also claimed a broad spectrum of activities from antibacterial to anti-inflammatory activities through analgesic, antiviral, anti-dermatophyte, anticancer, and hepatoprotective properties (Sobeh et al., 2018). Two recent reviews very briefly highlighted the chemical composition, traditional uses and biological activities of the plant (Harsha et al., 2021; Subbulakshmi et al., 2021).

The present research survey tends to summarize the traditional uses, chemical constituents, and pharmacological properties of extracts and compounds from *S. jambos* in one document as much information as possible about this plant, which has many biological properties. This work could provide a scientific basis for future study and necessary information for further investigations of new drug discovery.

## Taxonomy and Botanical Description

The genus *Syzygium* contains approximately 1,200–1800 species, the majority of which are flowering plants (Khalaf et al., 2021). Its taxonomy has been disputed for long with that of the genus *Eugenia* (Mabberley, 2017). As a result, species of the later have been ranged in the genus *Syzygium*. Amongst them, *S. malaccence, S. suborbiculare, S. paniculatum, S. aqueum, samarangense,* and *S. jambos* (Sobeh et al., 2016; Cock and Cheesman, 2018). *S. jambos* L*.* Alston, synonym of *Eugenia jambos*, is native to Reunion Island, Central America (Guatemal), and South-East of Asia, especially in Nepal, Indonesia, Philippines, and Malaysia. It has been naturalized in India and claims various vernacular names in different cultures including malabar plum, plum rose, rose apple, and water apple (Maskey and Shah, 1982; Morton, 1987; Avila-Peña et al., 2007).

**FIGURE 1:**
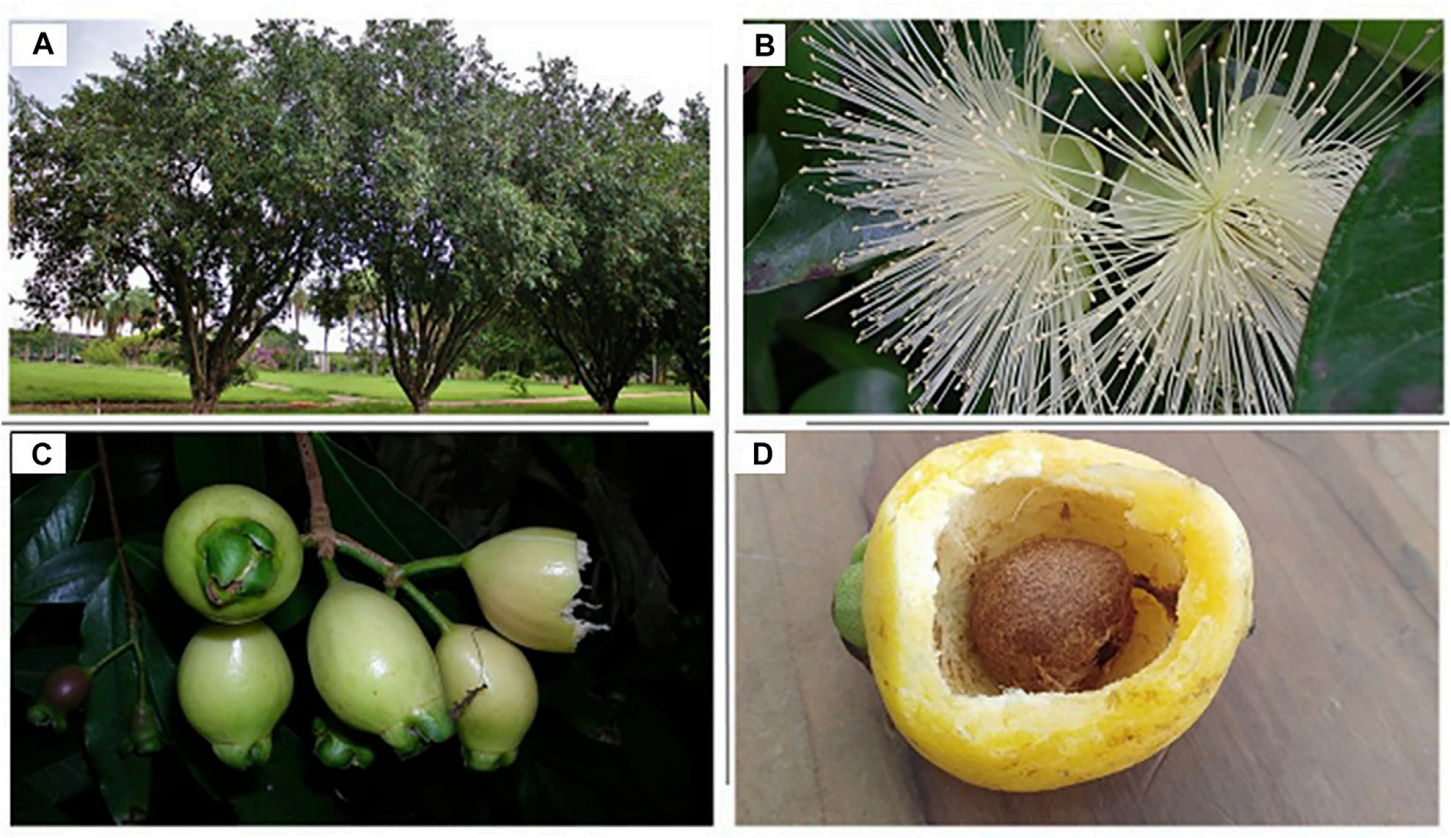
*Syzygium jambos* **(A)** tree, **(B)** flowers, **(C)** unripe fruits and **(D)** seeds.

*S. jambos* belonging to the family Myrtaceae*,* is a medium sized tree reaching 7.5–12 m in height, Figure 1 (Morton, 1987). Due to its physical characteristics and the aroma of the fruits, the plant is often known as rose apple. It has a dense crown of slender with wide spreading branches. Leaves are opposite, lanceolate, and glabrous with 2.5–6.25 cm wide and 10–22 cm length. They are glossy and dark-green when mature while vibrant red when young. Flowers are in small terminal clusters, white or greenish white with a diameter of 5–10 cm. Usually, there are 4–5 flowers together in terminal clusters (Nawwar et al., 2016). The berries have a fleshy pericarp with 10–15 mm thick on the tree. They are sub-globose and whitish-to pinkish-yellow color. Every fruiting season, a mature rose apple tree produces about 35.57 g of fruit, with 7.16 cm length and 5.15 cm width. The epicarp of the fruit is thin, smooth, and reddish, while the mesocarp and endocarp are whitish and succulent,Figure 1 (Daly et al., 2016; Mangini et al., 2020).

## Phytochemical Composition

**FIGURE 2:**
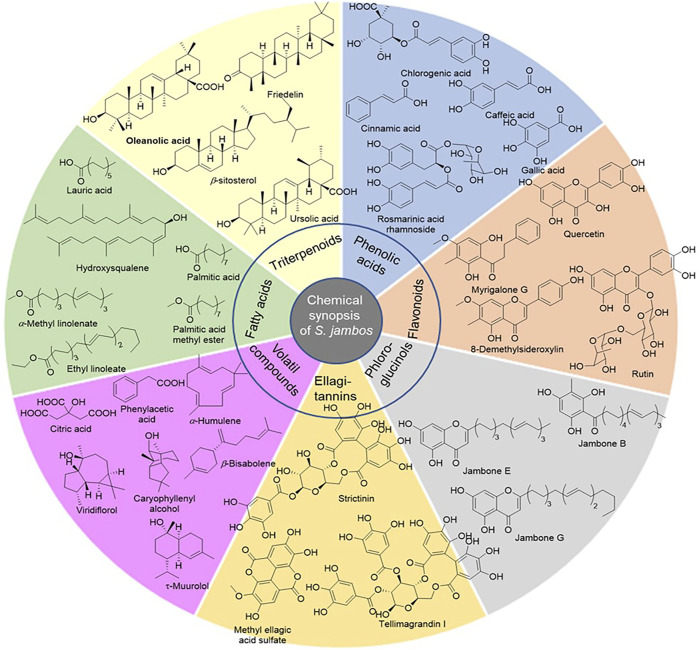
Selected secondary metabolites from *S. jambos*.

Phenolic compounds are mainly present in the leaves of *S. jambos*. They are represented by flavonoids, ellagitannins, phloroglucinols, and phenolic acids, Table 1; Figure 2 (Rocchetti et al., 2019; Slowing et al., 1994; Slowing et al., 1996; Sobeh et al., 2018). Flavonoids are the most abundant group of compounds while quercetin sounds to be the most abundant monomer in every organ of the plant, except the stem bark. It is found in both aglycone and saponin forms. Only flavone and chalcone-types of flavonoids occur in *S. jambos* (Reynertson et al., 2008). Some anthocyanidins have also been detected in the plant mainly, petunidin 3-*O*-glucoside, pelargonidin 3-*O*-(6″-malonyl-glucoside) and delphinidin 3-*O*-galactoside (Rocchetti et al., 2019). Catechin has been identified from the leaves of the plant suggesting a tentative occurrence of non-hydrolysable tannins in the plant. As part of tannins, only ellagitannins (hydrolysable tannins) have been found in some plant extracts to date. Likewise, ellagic acid monomer derivatives have also been reported in the leaves and stem bark of the plant. Moreover, phenolic acids, listed as intermediates in the metabolism of flavonoids and ellagic acids like gallic acid and cinnamic acid, have also been alarmed in the leaves and fruit of *S. jambos*. Gallic acid is the most abundant and distributed phenolic acid in the plant. The other phenolic acids were either glycosylated benzoic acid or derivatives of phenylpropanoids. Phloroglucinols also occur in *S. jambos* leaves. Though only one report highlighted their presence in *S. jambos*, phloroglucinols are well distributed in Myrtaceae family. The seven compounds of this class were isolated from a Chinese species and no trace of one of this group of compounds was mentioned in the Egyptian or Brazilian varieties, Table 1; Figure 2 (Li et al., 2015).

**TABLE 1:** Phytoconstituents from *S. jambos*.

Class of compounds	Compound names	Plant organs	Characterization methods	References
Flavonoids	Quercetin	Fruit, whole plant, leaves	HPLC, ESI-MS, EIMS, IR, 1D and 2D NMR	Slowing et al., (1994), Reynertson et al., (2008), Bonfanti et al., (2013), Hossain et al., (2016)
	Quercitrin	Fruit		Reynertson et al. (2008)
	Rutin	Whole plant		Ghareeb et al. (2017)
	5,4′-dihydroxy, 7-methoxy, 6-methyl-flavone			
	Isoetin-7-*O*-*β*-d-glucopyranoside			
	Myricetin 3-*O*-beta-d-xylopyranosyl (1->2) alpha-l-rhamnopyranosides	Leaves		Slowing et al. (1994)
	Kaempferol			Bonfanti et al. (2013)
	Quercetin 3-*O-*xylosyl-(1→2) rhamnoside	Whole plant		Nawwar et al. (2016)
	Quercetin 3-*O*-xylosyl- (1→2) xyloside			
	Quercetin 3-*O*-glucuronide			
	Myricetin 3-*O*-glucoside			
	Myricetin 7-methylether 3-*O*-xylosyl (1→2)rhamnoside			
	Myricetin 3′,5′-dimethyl ether 3-*O*-xylosyl (1→2)rhamnoside			
	Myrigalone B	Leaves		Jayasinghe et al. (2007)
	Phloretin 4 -*O*-methyl			
	Myrigalone G			
Triterpenoids	Oleanolic acid	Leaves		Li et al. (2015)
	Betulinic acid			
	Friedelin			Kuiate et al., (2007); Haque, (2015)
	3-nor-2,3-Secofriedelan	Stem bark, leaves		Haque, (2015)
	Β-Sitosterol	Stem bark		Lin et al., (2014); Haque, (2015)
	Β-Amyrin acetate			Kuiate et al. (2007)
	Lupeol			
	Ursolic acid			Lin et al. (2014)
	3-Acetyl-ursolic acid			
	Asiatic acid			
	Arjunolic acid			
	Morolic acid 3-o-caffeate			Ghareeb et al. (2017)
Phloroglucinol	Jambone A	Leaves		Li et al. (2015)
	Jambone B			
	Jambone C			
	Jambone D			
	Jambone E			
	Jambone F			
	Jambone G			
Ellagic acid and ellagitannins	Tellimagrandin	Leaves		Slowing et al. (1994)
	Limagrandin I			
	Strictinin			
	Casuarictin			Yang et al. (2000)
	2,3-hexahydroxydiphenoylglucose stachyurin			Slowing et al. (1994)
	Casuariin	Stem bark, leaves		
	3,3′,4′-tri-*O*-methylellagic acid	Leaves		Chakravarty et al. (1998)
	3,3′,4′-tri-*O*-methylellagic acid-4-*O*-β-d-glucopyranoside			
	1-*O*-galloylcastalagin			Yang et al. (2000)
	Castalagin	Stem bark, leaves		Sobeh et al., (2018); Mahmoud et al., (2021)
	Vescalagin			
	Phyllanthusiin G	Stem bark		Mahmoud et al. (2021)
	Ellagic acid pentoside			
	Ellagic acid			
	Methyl ellagic acid sulfate			
Phenolic acid	Gallic acid	Leaves, fruit	HPLC-PDA-MS/MS and GC-MS	Bonfanti et al., (2013), Nawwar et al., (2016)
	Cinnamic acid			Ghareeb et al. (2017)
	3,4,5-Trihydroxybenzoic acid			
	Prenylbenzoic acid 4-β-d-glucoside			
	4′-hydroxy-3′-methoxyphenol-β-d-[6- *O*-(4″-hydroxy-3″,5″-dimethoxylbenzoate)] glucopyranoside			
	Caffeic acid	Leaves		Bonfanti et al. (2013)
	Chlorogenic acid			
	Rosmarinic acid rhamnoside			Sobeh et al. (2018)
Organic acids	Citric acid	Leaves	GC-MS	
	Malic acid			
Volatile compounds	Phenylacetic acid			Khalaf et al. (2021)
	Hexanal			Musthafa et al., (2017); Reis et al., (2021)
	Geraniol			
	Citronellol			
	Hotrienol			
	(E)-cinnamyl alcohol			
	Β-phenylethyl alcohol			
	(E)-2-methyl-2-buten-1-ol			
	Linalool			
	(Z)-3-hexen-1-ol			
	3-phenylpropanol			
	(Z)-3-hexen-1-ol			
	Β-caryoplyllane			
	Α-humulene			
	Β-bisabolene			
	(e,e)-α-farnesene			
	Caryophyllenyl alcohol			
	Caryolan-8-ol			
	N-heneicosane			
	Viridiflorol			
	Ledol			
	Humulene epoxide ii 1			
	Epi-cedrol 2			
	Epi-α-muurolol			
	Trans-(ipp vc oh) sesquisabinene hydrate			
	4,8-α-Epoxy-caryoplyllane			
	Trans-caryophyllene			
	Σ-Cadinene			
	Τ-Muurolol			
	Neophytadiene			
	2-propen-1-one, 1-(2,6-dihydroxy-4-methoxyphenyl)-3-phenyl-, (e)-			
	4h-1-Benzopyran-4-one, 2,3-dihydro-5,7-dihydroxy-6,8-dimethyl-2-phenyl-, (s)-			Musthafa et al. (2017)
	1h-Benzoimidazole, 5-ethoxy-2-phenethylsulfanyl			
	2,3-Dihydro-2,4-diphenyl-1h-1,5-benzodiazepine			
	α -Tocopherol			
	[3-Deuterium)- α -tocopheryl methyl ether			Guedes et al. (2004)
Fatty acid	Lauric acid			
	Caproic acid			
	Hentriacontane			
	3-Pentadecylphenol (3-n-pentadecylphenol)			
	(e, e)-1,4,4-trimethyl-8-methylene-1,5-cycloundecadiene			
	Methyl (z)-5,11,14,17-eicosatetraenoate			
	4h-1-benzopyran-4-one, 2,3-dihydro-5,7-dihydroxy-2-phenyl-(S)			Musthafa et al. (2017)
	3.7,11,15-tetramethyl-2-hexadecen-1-ol			
	Hexadecanoic acid, methyl ester			
	Hexadecanoic acid			
	Hexadecanoic acid, ethyl ester			
	9,12-Octadecadienoic acid, methyl ester			
	9,12,15-Octadecatrienoic acid, methyl ester, (z,z,z)-			
	9,12-Octadecadienoic acid (z,z)-			
	8,11,14-Eicosatrienoic acid, (z,z,z)-			
	Ethyl linoleate			
	Octadecanoic acid, ethyl ester			
	Hexadecanoic acid, 2-hydroxy-1-(hydroxymethyl)ethyl ester			
	2,6,10,14,18,22-Tetracosahexaene, 2,6,10,15,19,23-hexamethyl			

Pentacyclic triterpenoids are also abundant in the plant especially in the leaves and stem bark. They belong to oleanane, ursane, lupane and friedelane subclasses. The major ones were betulinic acid and friedelin. Saponins of triterpenes have not yet been isolated except the readily available β-sitosterol glucoside, Table 1 (Kuiate et al., 2007; Li et al., 2015). Roots and flowers of the plant have not been investigated yet.

The essential oil of the plant leaves contain mostly volatile sesquiterpenes including δ-cadinene, cumaldehyde, β-himachalene, isocaryophyllene, and *β*-cedrene, Table 1 (Khalaf et al., 2021). Linalool is one of the essential oil markers in the identification of the plant fruit. Indeed, linalool, cinnamyl alcohol, and geraniol are the main volatile terpenes in the extracts. Differences were observed in the volatile aromatic composition of fruits from the Brazilian, Malaysian, and Egyptian species. Linalool was found as the main compound in the Brazilian fruits while 3-phenylpropyl alcohol (Z)-3-hexen-1-ol and (Z)- cinnamaldehydes were identified as major compounds in the Malaysian and Egyptian ecospecies (Vernin et al., 1991; Wong and Lai, 1996; Guedes et al., 2004; Ghareeb et al., 2017).

## Traditional Uses

Rose apple carries a long history as essential traditional medicine with a broad spectrum of application in various cultures. In India, the fruit tonic helps to improve brain and liver health while fruit infusions convey diuretic property (Morton, 1987). Moreover, the juices from macerated leaves in water were used as a febrifuge (Maskey and Shah, 1982). Dysentery is also alleviated by the seeds together with diarrhea, and catarrh. Furthermore, the flowers are assumed to relieve fever (Baliga et al., 2017). The infusion of the powdered leaves is beneficial to diabetes (Maskey and Shah, 1982). In South American cultures, the seeds have an anesthetic property whereas leaf decoction is applied to sore eyes, and used as diuretic, expectorant and to treat rheumatism (Maskey and Shah, 1982). The decoction of the bark is administered to treat asthma, bronchitis, and hoarseness (Maskey and Shah, 1982). The plant is also used to treat hemorrhages, syphilis, leprosy, wounds, ulcers, and lung diseases due to its potency to relieve fever and pains. In China, each plant organ is used to treat digestive tract and tooth pains (Mahmoud et al., 2021; Reis et al., 2021).

## Biological Activities

**FIGURE 3:**
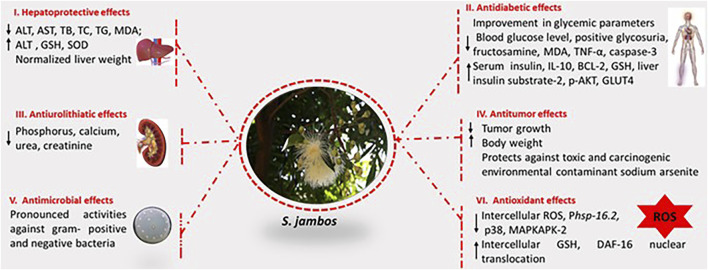
Biological activities of *S. jambos*.

The biological applications of *S. jambos* are rich and diverse. Isolates were screened in accordance with the traditional uses of the plant encountered worldwide. Mainly, plant extracts and compounds have presented antifungal, antibacterial, hepatoprotective, analgesic, antioxidant, anti-inflammatory, antidiabetic, anticancer, anti-pyretic activities, Figure 3. The main pharmacological characteristics of *S. jambos* are listed in Tables 2–4.

**TABLE 2:** Antimicrobial activity of *S. jambos* extracts.

Extract	Tested strains	Key results	Reference
Leaves			
Methanol extract	*Alcaligenes faecalis*	MIC = 797.5 µg/ml	Mohanty and Cock, (2010)
	A. Hydropilia	MIC = 384.6 µg/ml	
	*Bacillus* cereus	MIC = 182.6 µg/ml	
	*S. aureus*	MIC = 46.5 µg/ml	
	*Aeromonas* hydrophilia, *Citrobacter freundii*, *E. coli*, *Klebsiella pneumoniae*, *Proteus mirabilis*, P. fluorescens, *Salmonella* newport, *Serratia marcescens*, *Shigella* sonnei, S. epidermidis and *Streptococcus* pyogenes	These bacteria were not susceptible by *S. jambos* leaf extract	
Ethanolic extract	Chromobacterium violaceum DMST 21761	At 500 µg/ml, a highest inhibition in QS-dependent violacein pigment production was observed up to 90%	Musthafa et al. (2017)
	*P. aeruginosa* ATCC 27853		
Ethanolic extract	*P. aeruginosa*	At sub-MIC (500 µg/ml), the extract showed significant reduction in QS-regulated virulence determinants	Rajkumari et al. (2018a)
		The extract showed also 31.96% of decreases in biofilm formation of *P. aeruginosa*	
Ethanolic extract	P. acnes	MIC = 31.3 µg/ml	Sharma et al. (2013)
Hydroethanolic extract	*S. aureus*, *E. coli*, A. niger, C. albicans	*S. aureus*: MIC between 200 and 300 μg/ml	Donatini et al. (2013)
		No activity against *E. coli*, A. niger and C. albicans at 1,000 and 2000 μg/ml	
Decoction	P. vulgaris (ATCC 6896)	MIC = 31 μg/ml and MBC = 1.0 mg/ml	Luciano-Montalvo et al. (2013)
	S. saprophyticus (ATCC 15305)	MIC = 500 μg/ml and MBC = 2.0 mg/ml	
	*S. aureus* (ATCC 6341)	MIC = 500 μg/ml and MBC = 1.0 mg/ml	
Aqueous and methanolic extracts	C. albicans (ATCC10231)	IZ = 8–13 mm	Noé et al. (2019)
	Epidermophyton floccosum (ATCC 26072)	IZ = > 16 mm	
	Microsporum gypseum (ATCC7911)	IZ = 12.3 mm	
	Trichophyton mentagrophytes BSL2 (ATCC 13996)	IZ > 10 mm	
	Trichophyton rubrum (ATCC 22402)	IZ > 10 mm	
Ethanolic extract	*S. aureus*	Φmm = 20 mm	Khalaf et al. (2021)
	*E. coli*	Φmm = 8 mm	
	C. albicans	Φmm = 21 mm	
	A. niger	Φmm = 7 mm	
Acetone extract	*Staphylococcus aureus*	MIC = 128 μg/ml	Panthong and Voravuthikunchai, (2020)
85% MeOH	*S. aureus*, Methicillin-resistant, *S. aureus*, *P. aeruginosa*, C. albicans, and A. niger	Φ = 13.5, 11.0, 13.5, and 11.5 mm, respectively	Ghareeb et al. (2016)
Defatted 85% MeOH		Φmm ranging between 10 and 13.5 mm	
Petroleum ether		Φmm ranging between 8.5 and 11.5 mm	
Dichloromethane		Φmm ranging between 9 and 11.5 mm	
Ethyl acetate		Φmm ranging between 11.5 and 13.5 mm	
n-Butanol		Φmm ranging between 9.5 and 14.5 mm	
Aqueous		Φmm ranging between 12.5 and 15.5 mm	
Methanolic extract	26 strains of *S. aureus*	MIC ranging between 32 and 512 μg/ml	Wamba et al. (2018)
	*Enterobacter* aerogenes EA294	MIC = 64 μg/ml	
	*Enterobacter cloacae* (ECCI69)	MIC = 512 μg/ml	
	*Pseudomonas aeruginosa* (PA01, PA124)	MIC = 512 μg/ml	
	Providencia *stuartii* (NEA16, PS2636)	MIC = 128 and 256 μg/ml, respectively	
	*Klebsiella pneumoniae* K24	MIC = 64 μg/ml	
	*E. coli*	MIC range of 128 and 512 μg/ml	
**Bark, leaves and seeds**			
Acetone extract	*Staphylococcus aureus*	Φmm ranging between 7 and 12 mm	
Aqueous extract	*Bacillus* subtilis	Φmm ranging between 12 and 16 mm	Murugan et al. (2011)
	*Escherichia coli*	Φmm ranging between 6 and 17 mm	
	*Klebsiella pneumoniae*	Φmm ranging between 12 and 15 mm	
	*Proteus* vulgaris	Φmm ranging between 9 and 12 mm	
	*Pseudomonas aeruginosa*	Φmm ranging between 12 and 15 mm	
	*Salmonella typhi*	Φmm ranging between 8 and 12 mm	
	*Vibrio* cholera	Φmm ranging between 12 and 15 mm	
**Bark**			
Acetone and aqueous extracts	*S. aureus*	MIC ranged between 500 and 1,000 μg/ml	Djipa et al. (2000)
	Y. enterocolitica	MIC ranged between 250 and 750 μg/ml	
	S. hominis	MIC ranged between 15 and 250 μg/ml	
	S. cohnii	MIC = 250 μg/ml, in both extracts	
	S. warneri	MIC ranged between 15 and 750 μg/ml	
**Flower**			
85% MeOH	*S. aureus*, Methicillin-resistant, *P. aeruginosa*, C. albicans, A. niger	Φmm between 8.5 and 10.5 mm	Ghareeb et al. (2016)
**Seeds**			
Aqueous extract	Microsporum gypseum	IZ = 28.75 mm	Sakander, et al. (2015)
	Microsporum canis	IZ = 30.25 mm	
	*Candida* albicans	IZ = 16 mm	

**TABLE 3:** *In vitro* effects of *S. jambos* extracts.

Extract	Activity	Used method	Country	Effects	Reference
**Whole plant**					
**e**thanol extract	Antioxidant	DPPH and NO scavenging assay	South Africa	DPPH (IC_50_ = 14.10 µg/ml) NO scavenging assay (Low activity)	Twilley et al. (2017)
	Anti-inflammatory	COX-2		IC_50_ of 3.79 µg/ml	
	Cytotoxic	A375, A431, HeLa and HEK-293 cell lines		IC_50_ ranged between 56 and 198 µg/ml	
	Antiviral	Anti-herpes simplex virus type-1 assay		The extract exhibited potential anti-viral activity at 50.00 μg/ml	
				100% viral inhibition when tested at the highest viral dose	
**Leaves**					
Hydroethanol	Antioxidant	DPPH	Brazil	EC_50_ = 5.68 µg/ml	Donatini et al. (2009)
		MDA		IC_50_ = 0.17 µg/ml	
Methanolic extract	Anti-inflammatory	Hyaluronidase inhibition assay	India	60.80% inhibition at 1 µg/ml	Reddy et al. (2014)
	Antioxidant	DPPH assay		IC_50_ = 41 ± 1.8 µg/ml	
		Nitric oxide assay		IC_50_ = 63 ± 1.6 µg/ml	
		lipid peroxidation		IC_50_ = 48 ± 20 µg/ml	
Ethanolic extract	Antioxidant	ABTS	Bangladesh	IC_50_ = 57.80 µg/ml	Hossain et al. (2016)
Methanolic extract	Antioxidant	DPPH	Egypt	IC_50_ = 5.7 ± 0.45 µg/ml	Sobeh et al. (2018)
		FRAP		IC_50_ = 19.77 ± 0.79 mM	
Ethanolic extract	Anticancer	XXT	South Africa	IC_50_ < 60 µg/ml against the HeLa and A431 cell line	Twilley et al. (2017)
	Antiviral	Cytopathic effect (CPE) inhibition assay		Potential antiviral activity with 100% viral inhibition for both (10 and 100 TCID_50_) viral doses against HSV-1	
	Antioxidant	DPPH		IC_50_ = 1.17 ± 0.30 μg/ml	
Methanolic, hexane and dichloromethane extract	Antiviral	Plaque Reduction Assay	Thailand	At 100 µg/ml, extracts of hexane and dichloromethane exhibited HSV-1/HSV-2 inhibitory activity greater than 50% inhibition	Athikomkulchai et al. (2008)
70% aqueous acetone extract	Cytotoxicity	MTT assay	Taiwan	IC_50_ = 10.2 µg/ml strongest cytotoxic effect on human promyelocytic leukemia cells (HL-60)	Yang et al. (2000)
Methanol extract	Cytotoxicity	SRB assay	Egypt	At 100 µg/ml, the extract exhibited an increase of MCF-7 cell proliferation	Rocchetti et al. (2019)
85% MeOH	Antioxidant	Phosphomolybdenum assay	Egypt	538.20 mg AAE/g extract	Ghareeb et al. (2016)
Deffated 85% MeOH				619.51 mg AAE/g extract	
Petroleum ether				147.96 mg AAE/g extract	
Dichloromethane				222.76 mg AAE/g extract	
Ethyl acetate				460.15 mg AAE/g extract	
n-Butanol				643.90 mg AAE/g extract	
Aqueous				315.44 mg AAE/g extract	
Ethanolic extract	Antioxidant	DPPH	Bangladesh	IC_50_ = 14.10 μg/ml	Islam et al. (2012)
Methanolic and ZnO-NPs extract	Antiurolithiatic	Single diffusion gel growth technique	India	PI = 19.63–30.56% of inhibition at 2% of extract	Deka et al. (2021)
				PI = 16.28–24.68% of inhibition at 0.5% of extract for ZnO-NPs extract, PI = 25.60 at 0.5 and 35.27% at 5%	
Methanolic extract	Antioxidant	DPPH	Egypt	IC_50_ = 48.13 µg/ml	Khalaf et al. (2021)
Ethanolic extract	Antioxidant	DPPH	India	IC_50_ = 38.73 µg/ml	Rajkumari et al. (2018b)
Aqueous ethanolic extract	Antioxidant	DPPH	Egypt	EC_50_ = 13.52 ± 0.69 µg/ml	Nawwar et al. (2016)
		ORAC assay		EC_50_ = 34.35 ± 12.45 µg/ml	
	Cytotoxicity	Neutral red uptake assay		HaCaT (IC_50_ = 106.74 ± 10.89 µg/ml)	
				Bladder carcinoma cells (IC_50_ = 55.24 ± 2.67 µg/ml)	
**Fruit**					
Methanolic extract	Antioxidant	DPPH	United States	IC_50_ = 92.0 ± 8.24 µg/ml	Reynertson et al. (2008)
Hydroalchohlic extract	Antioxidant	DPPH	Pahang	IC_50_ = 24.44 µg/ml	Yunus et al. (2021)
Ethanolic extract	Antioxidant	DPPH	Malaysia	Lowest activity, IC_50_ = 24.44 μg/ml	
	Antidiabetic	α-Glucosidase inhibition assay		Low inhibition activity, IC_50_ = 0.67 ± 0.04	
n-Hexane, DCM and MeOH	Cytotoxicity	HeLa and Vero cell lines	Bangladesh	Not active	Nesa et al. (2021)
**Seed**					
Methanolic extract	Antioxidant	DPPH and ORAC	Brazil	112.06 and 489.62 µmol/g Trolox equivalent, respectively	Vagula et al. (2019)
Ethanolic extract	Antioxidant	ABTS	China	IC_50_ = 45.79 ± 1.02 µg/ml	Zheng et al. (2011)
		Hydroxyl radical activity		IC_50_ = 65.22 ± 0.93 µg/ml	
		DPPH		IC_50_ = 95.21 ± 1.78 µg/ml	
Flowers					
85% MeOH	Antioxidant	Phosphomolybdenum assay	Egypt	560.97 mg AAE/g extract	Ghareeb et al. (2016)

AAE: ascorbic acid equivalent; PI: percentage inhibition of the struvite crystals.

**TABLE 4:** *In vivo* effects of *S. jambos* extracts.

Extract	Doses	Route	Model	Activity	Country	Effects	Reference
**Aerial parts**							
Hydro-alcoholic	100–300 mg/kg	Intraperitoneal injection	Male Sprague–Dawley rats	Anti-inflammatory	Venezuela	Analgesic effect on inflammatory cutaneous and deep muscle pain	Ávila-Peña et al. (2007)
**Leaves**							
Hydroethanolic	400 mg/kg	Oral	Gastric injury induced by HCL/ethanol to rats	Anti-ulcerogenic	Brazil	Reduction of the subcronic ulcer	Donatini et al. (2009)
Ethanolic	400 mg/kg	Oral	Rats, induced with acute inflammation	Anti-inflammatory	Bangladesh	Acute anti-inflammatory activity	Hossain et al. (2016)
Methanolic	200 mg/kg	Oral	Rats, CCl_4_ acute induced hepatic injury	Hepatoprotective	Egypt	The extract decreased the levels of all measured liver makers, including ALT, AST, TB, TC, TG, and MDA, while increasing GSH and SOD.	Sobeh et al. (2018)
	200 μg/ml	Juglone induced oxidative stress	*Caenorhabditis elegan*s	Antioxidant		Decrease the intracellular ROS level in a dose dependent manner by 59.22%, the survival activity was also very low and dose dependent	
Methanolic	100–200 mg/kg	Oral	Paracetamol-induced hepatic damage in Wistar albino rats	Hepatoprotective	-	The extract cased a significant decrease in the serum hepatic enzyme levels, SGOT, SGPT, ALKP, and serum Bilirubin in dose-dependent manner	Selvam et al. (2013)
Ethanolic	300 mg/kg	Intraperitoneal injection/oral	Rats, CCl_4_ induced hepatic injury	Hepatoprotective	Bangladesh	Gradual normalization of serum markers enzyme (SGPT, SGOT, ALP), total bilirubin, total protein, and liver weight	Islam et al. (2012)
Methanolic	250 mg/kg	NS	Rats, Ethylene glycol-induced urolithiasis model	Antiurolithiatic	India	reduced the phosphorus, calcium, urea, and creatinine levels in the serum	Deka et al. (2021)
Ethanolic	500 μg/ml	NS	*S. cerevisiae* (wild type and mutant strain)	Antioxidant	India	H_2_O_2_ scavenging potential	Rajkumari et al. (2018b)
Decoction	220 mg/kg	Oral	C57BL/J ob/ob Mice	Hypoglycemic	Puerto Rico	Better blood glucose modulation over time	Gavillán-Suárez et al. (2015)
**Bark**							
Aqueous	100–200 mg/kg	Oral	Streptozotocin–induced diabetes in rats	Antidiabetic	Egypt	Protective effects against STZ-induced diabetes	Mahmoud et al. (2021)
						Improvement in glycemic parameters	
						Suppression of pancreatic oxidative stress, inflammation, apoptosis, and insulin signaling pathway in the liver	
**Fruit**							
Pectic polysaccharides	150, 250 mg/kg	Intraperitoneal injection	Mice bearing Ehrlich solid tumor	Antitumor	Brazil	Reduced tumor growth and improved the body weight of tumor bearing mice	Tamiello et al. (2018a)

Ns: Not specified.

### Toxicity Studies

To date, only few literatures have reported the toxicity of the plant. The leaf extract of *S. jambos* is safe at a dose up to 5 g/kg b.wt. assessed by the acute toxicity test (Dhanabalan and Devakumar, 2014). The toxicity of the methanol extract of *S. jambos* and its fraction were evaluated by shrimp lethality bioassay. Methanolic extract and carbon tetrachloride fraction displayed significant lethality with LC_50_ = 6.97 and 13.61 µg/ml, respectively. Whereas the chloroform and hexane fractions showed moderate to low lethality with LC_50_ = 64.94 µg/ml and 257.6 µg/ml, respectively (Haque, 2015). In the same line, Ghareeb et al. (2016) tested different extracts and fraction obtained from the leaves and flowers against the brine shrimp *Artemia salina*, a useful tool to determine the toxicity of natural products. As a result, the *n*-butanol fraction of the leaves showed a strong toxicity with LC_50_ = 50.11 µg/ml while the dichloromethane and petroleum ether fractions were less toxic (LC_50_ = 446.65 µg/ml) (Ghareeb et al., 2016).

Toxicology safety evaluation is essential for plants applications and new drug development. However, the toxicological studies of extracts and compounds isolated from *S. Jambos* have not been fully explored yet. Therefore, further research in toxicity is needed to determine the suitability of the plant extracts and related compounds composition.

### Antimicrobial Activity

Diverse antimicrobial activity of crude extracts and isolated compounds from the plant were described in previous reports. Disc diffusion assays, agar well diffusion, and broth microdilution procedures were employed to assess the antibacterial activity of plant extracts. As shown in Table 2. Microbial growth inhibition zones and percentages, as well as minimum inhibitory concentrations (MICs), demonstrated that *S. jambos* has potential as a significant antibacterial agent.

Wamba et al. (2018) reported the capacity of *S. jambos* extracts to increase the potency of chloramphenicol antibiotic towards bacteria strains expressing MDR phenotype (Wamba et al., 2018). Leaf and bark extracts of the plant expressed up to 70% of antibiotic-modulating activity against *S. aureus* strains at MIC/2. Similar results were obtained in association with tetracycline, ciprofloxacin, and erythromycin against Gram-negative bacteria including strains of *Escherichia coli* (AG100ATet, AG102), *Enterobacter aerogenes* (EA27, EA289), *Klebsiella pneumoniae* (KP55, KP63), *Providencia stuartii* (PS299645, NEA16) and *Pseudomonas aeruginosa* (PA01, PA124) (Wamba et al., 2018). Likewise, *S. jambos* leaf extracts demonstrated potent antiviral effects on the virus involved in vesicular stomatitis and against different types of herpes simplex virus (Abad et al., 1997; Athikomkulchai et al., 2008).

Isolated compounds friedelin, *β*-amyrin acetate, betulinic acid, and lupeol, from the bark extract, were tested for their antidermatophytic activity against three commonly dermatophyte species found in Cameroon namely *Microsporum audouinii*, *Trichophyton mentagrophytes* and *T. soudanense*. Betulinic acid and friedelolactone were the most active compounds with MIC ranging from 12.5 to 100 µg/ml and the most sensitive fungi were *Trichophyton soudanense* (MIC = 25 µg/ml) and *Trichophyton mentagrophytes* (12.5 µg/ml) (Kuiate et al., 2007). The phenolic compounds, quercetin, rutin, prenylbenzoic acid 4-*O*-*β*-D-glucopyranoside, morolic acid 3-*O*-caffeate, 5,4′-dihydroxy-7-methoxy-6-methylflavone, 3,4,5-trihydroxybenzoic acid, isoetin-7-*O*-*β*-D-glucopyranoside, and (4′-hydroxy-3′-methoxyphenol-*β*-D-[6-*O*-(4″-hydroxy-3″,5″-dimethoxylbenzoate)] glucopyranoside) also exhibited both antibacterial and antifungal potentials with a diameter of inhibition zones ranging from 9–19 mm (Ghareeb et al., 2017). Accordingly, the antimicrobial activity of *S. jambos* crude extracts have been related to the presence of an increased level of tannins in the preparation (Baliga et al., 2017).

Moreover, silver nanoparticles synthetized from leaves and bark extracts of *S. jambos* showed higher antiplasmodial activity against chloroquine sensitive and resistant strains of *Plasmodium falciparum* (Dutta et al., 2017). The fatty compounds, ethyl linoleate, methyl linolenate and phytol, inhibited the QS-dependent pigment production in *C. violaceum* and lowered pyoverdine production in *P. aeruginosa* as well. Results were also confirmed by docking analysis (Musthafa et al., 2017). The above research confirmed the antimicrobial activity of *S. jambos*. However, it is worthy to note that the above studies focused on the *in vitro* evaluations. Consequently, these studies only give preliminary information about the activity of *S. jambos*. Therefore, further studies combining *in vivo* and *in vitro* need to be conducted to provide reliable basis for exploring new potentially and low toxic antimicrobial agents from the studied plant.

### Antioxidant Activity

Several studies, both *in vitro* and *in vivo*, reported the antioxidant activity of *S. jambos* extracts and its phytochemicals. Bonfanti et al. (2013) demonstrated the potency of the leaf aqueous extract of *S. jambos* to inhibit the nitric oxide radical, the lipid peroxidation and the mitigation sodium-nitroprusside-induced oxidative stress in rats. The extract also showed a capacity to increase the GSH levels in rats (Sobeh et al., 2018). Furthermore, the bark extract inhibited lipid peroxidation and increased reduced glutathione (GSH) in pancreatic tissues of STZ-diabetic rats (Mahmoud et al., 2021). *S. jambos* leaf extract abolished ROS production by endothelin-1 in human polymorphonuclear and mononuclear cell migration (Inostroza-Nieves et al., 2021). On the other hand, *S. jambos* rich phenolic and flavonoid fractions demonstrated good antioxidant activities as shown in Table 3. The chalcones phloretin 4′-*O*-methyl ether, myrigalones B and G were assessed for their antioxidant activity using DPPH radical. As a result, myrigalone B showed a significant capacity of scavenging radicals with an IC_50_ of 3.8 µg/ml while the other compounds showed low to moderate activity (IC_50_ > 30 µg/ml) (Jayasinghe et al., 2007). Moreover, 2,6-dihydroxy-4-methoxy-3,5-dimethyldihydrochalcone showed anti-DPPH activity with an IC_50_ value of 10.6 µg/ml while, the flavones, 4′-methoxysideroxylin and 6-demethylsideroxylin, and phloroglucinols, jambones A-B, presented weak antioxidant activities in FRAP and DPPH radical scavenging activities (Li et al., 2015).

### Neurological Activity

There are relatively few studies on neuroprotective effect of *S. jambos*. Bonfanti et al. (2013) investigated the effects of *S. jambos* in the inhibition of both AChE and BuCE, the two main enzymes in the occurrence of Alzheimer. As a result, the aqueous leaves extract of S. jambos showed significant AChE (IC_50_ = 16.5 µg/ml) and BuCE (IC_50_ = 15.2 µg/ml) inhibition potentials in support with the uses of the plant to alleviate Alzheimer disorders. Considering these findings, further investigations may improve the neuroprotective effect of *S. jambos*.

### Anticancer Activity

*In vitro* anticancer activity of isolates from *S. jambos* was determined towards various cancer cell lines, providing data on the bioactivity of both extract and single compounds, Table 3 *.* Methanolic extract of *S. jambos* leaves showed cytotoxic effects against liver cancer cell line*,* Hep G2 cells, by inducing apoptotic pathways (Thamizh Selvam et al., 2016). Moreover, another study evaluated the anticancer effects of the leaves along with other extracts on human melanoma (A375), epidermoid carcinoma (A431), cervical epithelial carcinoma (HeLa) and human embryonic kidney cells (HEK-293). They found that the extract showed low toxicity against HEK-293 cells but better effects against A431 and HeLa cells (IC_50_ = 34.90–56.20 μg/ml) (Twilley et al., 2017). The hydrolysable tannins, 1-*O*-galloyl castalagin and casuarinin, exhibited significant cytotoxic activity against the human promyelocytic leukemia cell line HL-60 with IC_50_ of 10.8–12.5 µM and showed moderate to low cytotoxicity on the human adenocarcinoma SK-HEP-1, normal cell lines of human lymphocytes and liver cell lines. Results were confirmed by DNA fragmentation assay and microscopic investigation of cells (Yang et al., 2000). The cytotoxic effects of the phenolic compounds, *cis*-3-*p*-coumaroylalphitolic acid and 4′-methoxysideroxylin, on melanoma SK-MEL-28 and SK-MEL-110 cell lines were assessed as well as that of the normal Vero cells, following the MTT assay. The compounds, displayed potent effects on the two melanoma cells with IC_50_ ranging from 18.3–81.5 µM (Li et al., 2015). The cytotoxic effect of quercetin-3-*O*-*β*-D-xylofuranosyl-(1 → 2)-*α*-L-rhamnopyranoside and myricetin-3-*O*-*β*-D-xylofuranosyl-(1 → 2)-*α*-L-rhamnopyranoside isolated from the CH_2_Cl_2_/MeOH fraction of the plant was evaluated against RW 264.7 cell lines. Both flavonoids demonstrated a moderate activity (IC_50_ = 1.68 and 1.11 µM, respectively) (Ticona et al., 2021). The cytotoxic effect of the nanoparticles synthetized from the leaf and bark extracts of *S. jambos* was assessed against HeLa and L6 cells using MTT assay. As a result, the nanoparticles were found to be non-toxic toward HeLa and L6 cell lines (Dutta et al., 2017). These investigations provided the anticancer potential of *S. jambos,* further *in vivo*, toxicological, and clinical studies are needed in future to guarantee efficiency and safety.

### Anti-Inflammatory Effect

Inflammation and specifically low-grade inflammation play a vital role in many diseases. Natural products with anti-inflammatory effects are promising targets for drug discovery. *In vitro* and *in vivo* models were applied to determine the anti-inflammatory effects of crude extracts and pure compounds from *S. jambos. In vitro* studies showed that the ethanol leaf extract of *S. jambos* and the commercially available chemicals ursolic acid and myricitrin dramatically reduced the release of inflammatory cytokines IL 8 and TNF-α by 74–99% indicating anti acne effects (Sharma et al., 2013). A more recent study on two isolated glycosylated flavonoids, the quercetin-3-O-β-D-xylofuranosyl-(1 → 2)-α-L-rhamnopyranoside and myricetin-3-O-β-D-xylofuranosyl-(1 → 2)-α-L-rhamnopyranoside, isolated from the chloroform/methanol fraction of *S.* jambos showed that they reduced the production of TNF-α, with IC_50_ values of 1.68 and 1.11 M, respectively in the RAW 264.7 cell line. In addition, at a dose of 5 mg/kg, the flavonoids reduced the levels of TNF-α, C-reactive protein, and fibrinogen in murine models (Apaza Ticona et al., 2021). *In vivo* studies showed that the ethanol extract of the leaves also exerted potent anti-inflammatory effects at a dose of 400 mg/kg in carrageenan and histamine edema rat models (Hossain et al., 2016). The soluble fraction of polysaccharide fraction of the plant also expressed a capacity to increase the secretion of TNF-α, IL-1β and IL-10 in a concentration-dependent manner (10–100 µg/ml). The aqueous extract of the plant attenuated the inflammatory response induced by LPS at a concentration of 100 µg/ml (Tamiello et al., 2018b). Furthermore, the bark extract inhibited pancreatic inflammation in STZ diabetic rat model where it dose-dependently suppressed the pro-inflammatory, TNF-α and increased the anti-inflammatory IL-10 levels (Mahmoud et al., 2021).

### Hepatoprotective Activities

Liver is one of the largest and important organs in human body and performs numerous interrelated vital functions, such as metabolism, biotransformation, and detoxification of toxins. Consequently, liver diseases resulting from liver damage is a global problem. Herbal medicine has been used traditionally for the prevention of liver diseases (Islam et al., 2012). Preclinical studies have shown that extracts from different parts of *S. jambos* possess beneficial effect in liver related diseases, Table 4. The methanol extract of the leaves of the plant significantly modulated the levels of liver biochemical parameters ALT, AST, MDA, TB, TC, TG, GSH and SOD) in comparison with the positive control, silymarin, Table 4 (Sobeh et al., 2018). Isolation of the compounds of the extract may led to the discovery of promising active constituents.

### Antidiabetic Activity

Diabetes and diabetic complications are global health problem. Although many medicinal plants were investigated for their possible antidiabetic activities, there are relatively few studies on antidiabetic effect of *S. jambos* extracts. An *in vitro* study compared the inhibitory effects of ethanol extract of different organs of *S. jambos* on α-glycosidase and α-amylase activities, enzymes related to diabetes, and showed that the inhibitory effects against yeast and mice intestinal α-glucosidase activity was on the following order: seed ˃ stem ˃ leaf ˃ root ˃ flower ˃ flesh ˃ acarbose, while the inhibitory effect on α-amylase activity was acarbose ˃ seed ˃ stem ˃ root ˃ leaf ˃ flesh ˃ flower (Wen et al., 2019). *In vivo* studies showed that the infusion of the combined leaves of *S. jambos* and *S. cumini* had no significant effect on blood glucose levels in a randomized double-blind clinical trial in non-diabetic and diabetic subjects (Teixeira et al., 1990). However a more recent study showed that the ethanol extract of leaves at two dose levels (374.5 mg/kg and 749 mg/kg, Po) lowered blood glucose levels in alloxan induced diabetic rabbits (Prastiwi et al., 2019). Moreover, an aqueous leaf extract from the plant showed better blood modulation potential of glucose over time, in diabetes genetic mouse models (Gavillán-Suárez et al., 2015). Recent studies have shown the protective effect of the bark extract on pancreatic β cells against streptozotocin-induced diabetes. The extract have also improved insulin signaling pathway in the liver and glycemic parameters and have suppressed pancreatic oxidative stress (Mahmoud et al., 2021). However, further studies need to be conducted to confirm the potential of *S. jambos* as a natural antidiabetic agent, as it can be incorporated into functional foods and nutraceutical products.

### Antiurolithiatic Activity

The antiurolithiatic activity of the leaf extract of *S. jambos*, collected in India, was evaluated both *in vitro* and *in vivo* using ethylene glycol induced urolithiatic model in rats. Results showed a capability of the extract to prevent the growth of urinary stones. However, further studies should be done to understand the mechanism and pharmacological action in preventing urolithiasis in susceptible populations (Deka et al., 2021).

## Discussion

The main chemicals found in *S. jambos* were phenolic compounds and triterpenoids. Phenolic compounds were the major constituents of the plant. They are made up of glycosylated flavonoid and ellagitannin derivatives. Plant extracts showed significant antibacterial activity, improving the potency of strong antibiotics like tetracycline, ciprofloxacin, erythromycin, or chloramphenicol. Likewise, both water-soluble fraction and organic extracts have shown significant capabilities in reducing radicals and heavy metal ions. *In vivo* anti-inflammatory activity of plant extracts has also been demonstrated with considerable endpoints. These biological characteristics of the plant could be related to their main chemical constituents. Flavonoids and ellagitannins are excellent free radical scavengers (Koagne et al., 2020). For this reason, they protect cells from aging and stress, and exerted anti-nociceptive activities. Indeed, *S. jambos* plant extracts have shown considerable anti-inflammatory activity towards some models. The analgesic potential has been ascribed to two glycosylate flavonols occurring in rose apple namely, myricetin-3-*O*-*β*-D-xylofuranosyl-(1 → 2)-*α*-L-rhamnopyranoside and quercetin 3-*O*-*β*-D-xylopyranosyl-(1→2)-*α*-L-rhamnopyranoside. However, no mechanism of action of the recorded biological activity was proposed yet. Nevertheless, both antioxidant and anti-inflammatory activities encountered for *S. jambos* extracts and compounds are closely related. The anti-inflammatory potency of rose apple extracts is a key point in the uses of plant extracts to alleviate different illnesses. More importantly, the major constituents of *S. jambos* extracts, flavonoids and ellagitannins, are mostly glycosylated. They can then be found in large extent in the blood because of their water solubility. This parameter is quite important in drug development as it improves the therapeutic action of a drug. Accordingly, *S. jambos* constitutes a potential candidate to the development of potent traditional drugs against ROS and inflammation-induced illness.

## Conclusion and Perspectives

This review provides an up-to-date summary of *S. jambos* from the perspectives of its phytochemistry, pharmacology, traditional uses as well as toxicology. Phytochemical investigations have been focused on different organs of the plant, prepared with various organic and water solvents. These studies revealed the presence of flavonoids (flavones, chalcones, anthocyanins and proanthocyanins), ellagitannins, phenolic acids, triterpenoids, volatiles compounds and fatty analogues. Compounds were either isolated following chromatographic techniques or identified by online methods like HPLC-MS/MS and GC-MS. Flavonoids and saponins as well as phenolic acids are the main constituents of the plant.

Activities of the plant towards pathogens and cells are also diverse and rich, consecutive to the broad spectrum of applications of the plant in traditional medicine to alleviate some illnesses. Plant extracts showed considerable anti-inflammatory activity and a synergistic effect to antibiotics activity of some popular drugs correlating the uses of the plant to relieve pains and infection. Extracts have also antiviral, anti-dermatophyte, hepatoprotective, and anticancer effects. Numerous compounds were isolated and initially screened for their bioactive potential. Further investigations are needed to complete the phytochemical profile, pharmacology mechanisms and pharmacokinetics studies of the plant. In the same line, toxicity study of *S. jambos* is indispensable in the future to assess the safety of the plant and its bioactive compounds to support possible future medicinal applications and before proceeding to the development of pharmaceutical formulations.

## Abbreviations

A375, Human melanoma cancer cell line; A431, Epidermoid carcinoma cancer cell line; AChE, Acetylcholinesterase; ALA, *Artemia* lethality assay; BuCE, Butyrylcholinesterase; COX-2, Cyclooxygenase-2 inhibition assay; DNA, Deoxyribonucleic acid; HEK-293, human embryonic kidney cells; HeLa, Cervical epithelial carcinoma; L6, Rat skeletal muscle cell line; MCF-7, Human breast cancer cell line; MDA, Malondialdehyde; MDR, Multidrug resistance; MIC, Minimum inhibitory concentration; MTT, 3-(4,5-dimethylthiazol-2-yl)-2,5-diphenyl-2H-tetrazolium bromide; ROS, Reactive oxygen species; SRB, Sulforhodamine-B; WSP, Water soluble polysaccharides; XTT, 2,3-Bis-(2-methoxy-4-nitro-5- sulfophenyl]-2Htetrazolium-5-carboxyanilide salt; ZOI, Zone of infection.
